# Underutilisation of rehabilitation for children with cancer in Gaza: the experience of the non-governmental organisation Children Not Numbers

**DOI:** 10.3389/fped.2026.1844813

**Published:** 2026-07-14

**Authors:** Abu Sidhanee, Ibtisam Abo Jabal, Ilham Lubbad, Soha Nassar, Mahmoud Jamal, Jehad Alawna, Safiyyah Abbas, Trish Scanlan, Ala Shatali

**Affiliations:** 1 Children Not Numbers, London, United Kingdom; 2 Department of Physiotherapy, Al Aqsa Hospital, Gaza City, Palestine; 3 Azhar University of Gaza, Gaza City, Palestine; 4 Department of Physiotherapy, Al Rantisi Hospital, Gaza City, Palestine; 5 Department of Physiotherapy, Augusta Victoria Hospital, Jerusalem, Palestine; 6 The Children’s Hospital at Westmead, Sydney, NSW, Australia; 7 Department of Physiotherapy, Al Shifa Hospital, Gaza City, Palestine

**Keywords:** armed conflict, Gaza, oncology, paediatric cancer, paediatrics, physical activity, rehabilitation, war

## Abstract

**Introduction:**

The conflict in Gaza has severely affected children there, as well as healthcare, rehabilitation and paediatric cancer services. There is limited literature on how conflict impacts rehabilitation and physical activity for children with cancer, and our report explores the underutilisation of rehabilitation for children with cancer in Gaza.

**Context:**

Each year, 210 children are diagnosed with cancer in Gaza. Due to inadequate healthcare facilities, many cancer patients frequently sought care outside the territory. Due to Israeli military attacks, Gaza’s only paediatric hospital and its paediatric cancer department were forced to close. Most rehabilitation facilities and vital infrastructure in Gaza have been damaged or destroyed, with many of the rehabilitation workforce displaced, injured, or killed.

**Programme:**

The non-governmental organisation Children Not Numbers was established in response to the conflict and provides health services, including rehabilitation, to children in Gaza. Over an 18-month period, sixty-seven children in Gaza with suspected or confirmed cancer were registered with CNN, many of whom exhibited symptoms such as pain, fatigue, and weakness. Despite these symptoms and impairments where rehabilitation could have been considered, none of this cohort was referred to the CNN rehabilitation team.

**Discussion:**

Globally, utilisation of rehabilitation services is generally low. Most of our cohort of children with cancer faced diagnostic and treatment challenges, and other factors that likely contributed to underutilisation of rehabilitation, including inadequate analgesia, undernutrition, conflict-related environmental risks, medical evacuation and other psychological and social difficulties.

**Conclusion:**

Protecting health and rehabilitation infrastructure and workforce is of immediate priority, alongside the swift, unobstructed evacuation of children with cancer, in accordance with international humanitarian law. With one in five of the world’s children living in conflict zones, it is vital to better understand the rehabilitation and physical activity needs of children with cancer in these areas.

**Limitations:**

Literature on rehabilitation and physical activity needs of children with cancer in conflict settings is scarce. Our report may omit other rehabilitation issues in Gaza, as children with suspected or confirmed cancer were not referred to or assessed by the CNN rehabilitation service. CNN’s experience may not be generalisable to other contexts or organisations.

## Introduction

The conflict in Gaza has had a devastating impact on children, including those with cancer. Gaza’s health care infrastructure and workforce have been significantly affected by the conflict, including rehabilitation and paediatric cancer services. In response, organisations such as the non-governmental organisation (NGO) Children Not Numbers (CNN) provide support to optimise the care of affected children in Gaza, including access to rehabilitation services.

Cancer affects various aspects of function and quality of life due to the disease itself and the long-term effects of treatment, which can persist well beyond treatment (1, 2). Two-thirds to 75% of all childhood and adult cancer survivors develop at least one long-term adverse effect that can impair function, participation, and quality of life (3, 4).

Paediatric rehabilitation supports children with functional impairments (5) and is often essential for managing the physical and psychological sequelae of cancer (6). Physical activity interventions for children with cancer enhance muscle strength, flexibility, fitness, body composition, and health-related quality of life (7). Rehabilitation and physical activity are crucial for reducing disability and optimising functional outcomes in the treatment of children with cancer (7) and should be regarded as a standard component of cancer care (8).

Rehabilitative services in paediatric cancer care are defined as healthcare services aimed at improving and restoring functional ability and quality of life for individuals with physical impairments and disabilities (9). Interdisciplinary teams providing rehabilitation and physical activity to children with cancer include physiotherapists, occupational therapists, speech and language therapists, exercise professionals, dietitians, rehabilitation doctors, nurses, prosthetists, and orthotists (4).

Although the literature discusses the impact of conflict on many aspects of cancer care, there is a notable lack of analysis on how conflict influences rehabilitation and health-related physical activity, as well as local rehabilitation systems and infrastructure, for children with cancer.

Our report seeks to explore the context of the rehabilitation needs specific to children with cancer in conflict zones, focusing on CNN’s work in Gaza.

## Context

Gaza, which is part of the Occupied Palestinian Territories (OPT) alongside the West Bank and East Jerusalem, had a population of 2.2 million before the current conflict, including over one million children (10, 11).

Following the attacks carried out by Hamas in Israel on October 7th, 2023, Israel’s military response in Gaza for the past two and a half years – determined by many as a genocide (12–14) – has included widespread attacks on healthcare and rehabilitation facilities and staff (15, 16), and a blockade of medical supplies (15, 17). The authors refer to the *current conflict* as the events during this timeframe, up to the time of publication.

Children are particularly vulnerable during armed conflict (18), and since half of Gaza’s population is under 18 (19), children have been disproportionately affected by the devastation. Conservative estimates indicate over 20,000 children have been killed by Israeli forces in Gaza—more than one every hour (20) —exceeding any conflict in the last twenty years (21). This figure does not include the number of children who have died from non-violent but related causes, including through the lack of healthcare, nutrition and shelter. Additionally, over 42,000 children have been injured (20), equating to 70 daily at points (22), though both child death and injury official figures are thought to be a significant underestimate (22, 23).

Despite the announced ceasefire in October 2025, conflict-related mortality and morbidity persist due to ongoing Israeli military attacks, with over 100 children killed from October 2025 to January 2026 (24). Approximately 3,800 children still require urgent medical evacuation, and rehabilitation capacity in Gaza remains critically limited (25).

Each year, 210 children are diagnosed with cancer in Gaza (26). The only paediatric cancer department in Gaza was located at Al-Rantisi Children’s Hospital (27). Due to the unavailability of some treatments, including radiotherapy (15, 28) and medication, and Gaza’s under-resourced healthcare facilities preceding the current conflict, both adult and paediatric cancer patients were forced to seek care outside Gaza (28, 29). Cancer care was the leading cause of referral outside Gaza during 2019–21, with children accounting for 21% of these referrals (30). Permit dependent, many previously travelled to Augusta Victoria Hospital (AVH) – Palestine’s only dedicated cancer centre, located in East Jerusalem, hospitals in Israel, or further abroad for cancer treatment (26, 28, 31).

The situations described are not limited to the current conflict (32), and indeed, many issues in Gaza’s health and cancer care systems are longstanding and predate it (27, 28). Past conflicts, the nearly 20 years of blockades and ongoing sieges have worsened gaps in childhood cancer care in Gaza, created shortages of cancer and palliative care medicines, and delayed children with cancer’s access to life-saving services, such as radiotherapy (33). The 2022 World Health Organisation (WHO) Director-General report on health conditions in Palestine (34) highlighted lower life expectancy, higher infant mortality, fragmented healthcare systems, permit delays and denials, attacks on health facilities and the killing of healthcare workers, amongst many issues.

Paediatric cancer care is severely disrupted by armed conflict, with ongoing military operations, blockades, movement restrictions and damaged health facilities leading to delayed diagnosis, medication shortages, inadequate follow-up care and delayed evacuation of critically unwell children (26). This has resulted in greater disease advancement, critical illness requiring intensive care, and mortality in Gazan children with cancer (26). Previous studies have highlighted the devastating impact of war on cancer care infrastructure and services, including the inability to maintain vital equipment, deterioration of services, limited and makeshift facilities, the expulsion of medical and health staff, populations cut off from cancer care, and ultimately the cessation of oncology services and care due to bombing and the destruction of oncology facilities (35).

The current conflict has created a catastrophic health reality for people with cancer in Gaza (36), with doctors reporting that cancer-related deaths have tripled since the conflict began (29). Gaza’s only paediatric hospital and its paediatric cancer department were forced to close due to Israeli military attacks (37), and many children with cancer are unable to leave the territory to seek treatment elsewhere (29) due to the protracted and unpredictable Israeli permit system, where rejections are common (28). Travel between Gaza and the West Bank, and consequently AVH, has also been shut off. Apart from one patient, following a court order in January 2026, no medical evacuations to the West Bank or East Jerusalem have been permitted by Israel since the start of the current conflict (38), significantly reducing access to care centres from which children may previously have received vital cancer treatment.

Conventional paediatric cancer diagnosis is often impossible due to limited cancer-specific diagnostics and imaging in Gaza. All of Gaza’s magnetic resonance imaging (MRI) scanners have been destroyed (39), leaving it without diagnostic MRI capabilities, while doctors report the unavailability of even basic diagnostic tools, such as biopsy needles (29). Many cancer patients remain without treatment as the entry of chemotherapy and drugs has been restricted (29), with chemotherapy supplies already exhausted in November 2023 (40). Even since the partial ceasefire, many children with cancer have died whilst awaiting Israeli permits to facilitate evacuation (41).

St Jude Global, a United States-based hospital which aims to improve access to care for children with cancer across the world (42), has been leading efforts to evacuate children with cancer to regional and international centres, and Jordan has taken hundreds of children through this programme. CNN is also supporting evacuation pathways for these children. These medical evacuations highlight the importance of collaboration in health care in conflict zones (26); however, according to WHO figures, only 250 children with cancer have been medically evacuated from the start of the conflict until late April 2026 (43).

Palestine was among 67 countries working to improve access and quality of care for children with cancer through the WHO-St. Jude Global Initiative for Childhood Cancer CureAll Framework, which recognises rehabilitation services as a key component of multidisciplinary care (44).

The conflict has also severely disrupted rehabilitation services. Israeli attacks have damaged or destroyed Gaza’s rehabilitation facilities and infrastructure. Rehabilitation services are unable to address the overwhelming demand (16), with fewer than one-third currently providing any services and none fully operational (15). Gaza previously had approximately 1,300 physiotherapists and 400 occupational therapists, but by September 2024, at least 42 had been killed (45). Additionally, much of the rehabilitation workforce has been injured and displaced (15, 16, 45).

While some rehabilitation-related professional groups, such as physiotherapists, were common in Gaza’s health care system, others, such as occupational therapists, were scarcer. Paediatric specialisation was rare, and most children, including those with cancer, received care from providers working with both adults and children. There were no dedicated paediatric oncology rehabilitation facilities, so patients from many speciality groups were treated by providers with general skills and experience (personal communication, Children Not Numbers, Gaza Rehabilitation Lead. November, 2025).

## Children Not Numbers (CNN)

In response to the current conflict, CNN was established as a non-governmental and charitable medico-legal organisation dedicated to improving the lives of Gaza’s children through a comprehensive approach that includes health, education, and rehabilitation. CNN has over 240 multidisciplinary team (MDT) members from more than 23 countries (46). The CNN rehabilitation team comprises international rehabilitation professionals who work remotely with a Gaza-based team to provide rehabilitation support for children.

The CNN MDT refer children needing rehabilitation services to the CNN rehabilitation team, including those with neuro-disability and traumatic injuries. The rehabilitation team operate through a network of clinicians and caseworkers who engage with families, both in person and remotely, to conduct rehabilitation assessments, discuss intervention goals, and recommend therapy programs and equipment to improve functional independence and quality of life. Gazan rehabilitation providers conduct face-to-face assessments and interventions when safe and practical, including for children displaced in refugee camps, while overseas rehabilitation providers develop rehabilitation programs and other treatment recommendations. This service ensures that many children, who would not be seen within the overstretched health system and cannot afford private healthcare, receive vital rehabilitation.

The CNN rehabilitation team uses broad referral criteria, accepting any child (aged up to 18 years of age) with rehabilitation-related issues or symptoms, or those who would benefit from assessment based on their diagnosis or current presentation. These include acute and new cases, as well as chronic problems that predate the current conflict. Patients with various medical and surgical issues, including orthopaedic, neurological, musculoskeletal and respiratory, can be referred, with the most frequent referrals being for war-related traumatic injuries, such as brain injury and limb loss and for children with pre-existing neurodisability, such as cerebral palsy. Children with cancer, with symptoms such as pain, weakness and fatigue, can be considered for rehabilitation input. In practice, decisions to refer to a specialist team, such as rehabilitation, are made by CNN MDT clinicians based on the information available about the child. Conflict zones can significantly negatively impact this, such as when medical records are destroyed by military attacks or lost during forced displacement.

Sixty-seven children in Gaza with a suspected or confirmed cancer diagnosis were registered with CNN over an 18-month period from April 1st, 2024, to October 1st, 2025. Thirty-eight (57%) of these children were male, and 29 (43%) were female, with a median age of 6 years (72 months). Twenty-eight (42%) children were aged 5 years or under.

**Figure 1:**
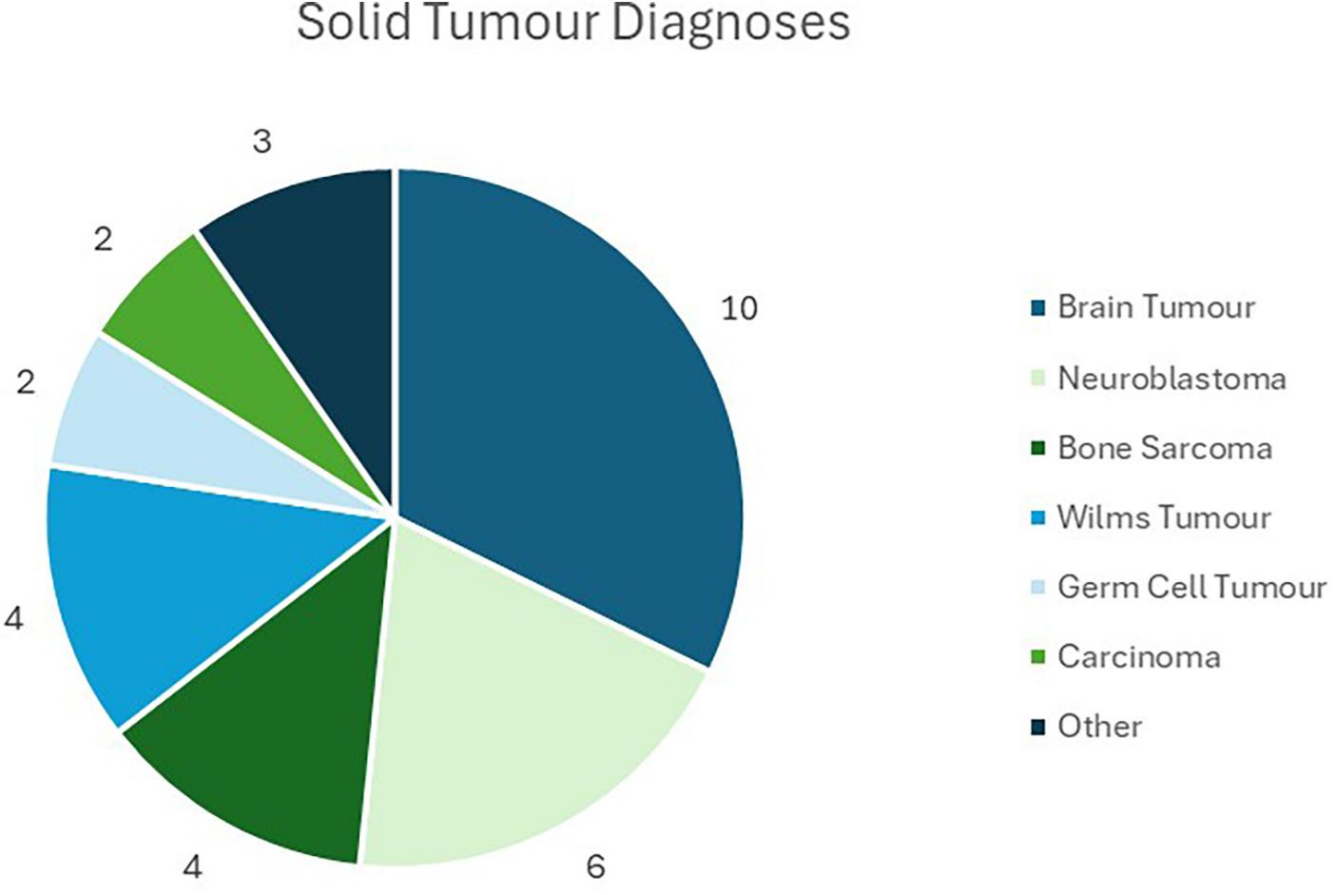
shows the breakdown of solid tumour diagnoses among children registered with CNN. Numbers around the chart indicate the number of children diagnosed with each solid tumour, totalling 31 children.

**Figure 2:**
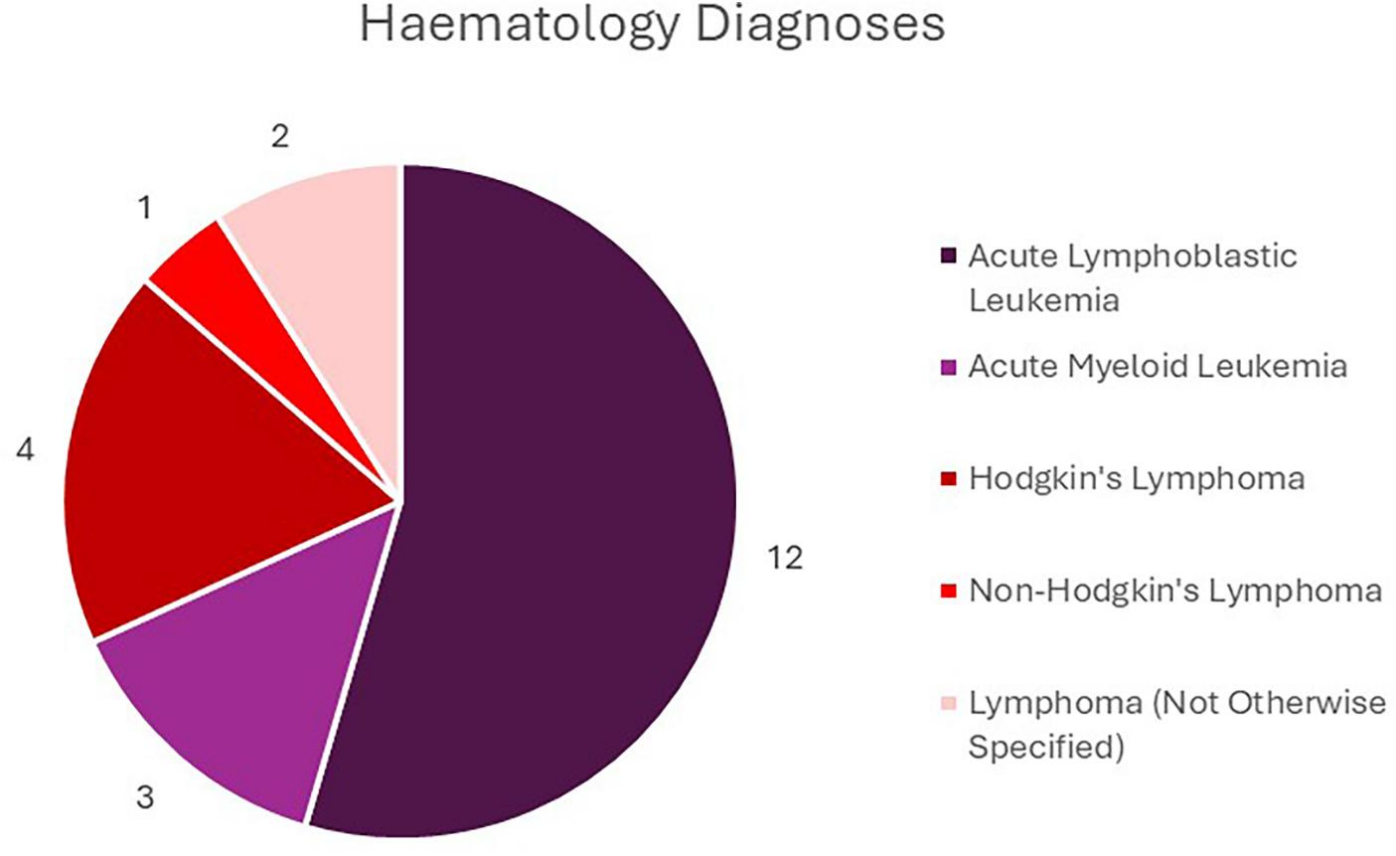
shows the breakdown of haematological malignancy diagnoses among children registered with CNN. Numbers around the chart indicate the number of children diagnosed with each haematological malignancy, totalling 22 children.

Forty-six per cent (*n* = 31) of this cohort of children presented with solid tumours, with the most common presentation being brain tumours (32%, *n* = 10), followed by neuroblastoma (19%) (Figure 1). Thirty-three per cent (*n* = 22) of the children had haematological malignancies, with leukaemia accounting for 68% of all haematological diagnoses, and lymphomas the remainder (Figure 2). The most common cancers in our cohort were acute lymphoblastic leukaemia (ALL) and brain tumours, which is consistent with a study conducted at AVH between 2018 and 2024 (31). Twenty-one per cent (*n* = 14) of children were diagnosed with presumed cancer based on clinical symptoms when diagnostic tests were not available. Half of these children (*n* = 7) were presumed to have haematological cancers, and the other half solid tumours.

Despite the CNN rehabilitation team having received over 200 referrals for other children with rehabilitation needs in Gaza, none of the 67 children with cancer were referred, despite documented symptoms and impairments where rehabilitation interventions could have been considered. Some children accessed rehabilitation services elsewhere, but this was not consistently documented.

Based on the limited records available to us, many of these children had a rehabilitation or physical symptom or needs, such as ‘pain’, ‘fatigue’, ‘general weakness’, ‘can no longer walk’, or ‘needs physiotherapy’, documented in their medical notes.

Many of these children would likely have benefited from a rehabilitation assessment and therapy directed towards identified functional impairments or developmental delay based on their diagnosis or age group, such as the 28 children with cancer in the early years age group (aged five years or under).

Pain was a common symptom experienced by this cohort of children with cancer. Cancer-related pain can be caused by multiple factors, including the disease itself, side effects or secondary complications of chemotherapy and other diagnostic or treatment-related procedures (47). In Gaza, many children with cancer cannot access basic pain relief (29), and analgesia is being rationed because of limited supplies (48).

Two children with cancer had a comorbid condition which would also have benefited from rehabilitation input. One child with cancer had a long-standing neurological condition, while another with cancer sustained significant war-related explosive injuries with impacts on mobility.

Medical records of this cohort also showed that 50% presented with severe or moderate acute malnutrition or were at risk of malnutrition.

Six children, who represent half of all non-evacuated paediatric cancer cases, died because of their cancer or cancer-related complications before evacuation. At the time of writing, 84% (*n* = 56) of this cohort had been evacuated.

## Discussion

### Underutilisation of rehabilitation services

The fact that none of the 67 children with cancer registered with CNN were referred to the CNN rehabilitation team is noteworthy and suggests that rehabilitation services were underused and/or not prioritised during the current conflict in Gaza.

Underutilisation of rehabilitation is an acknowledged phenomenon globally (49), with studies also demonstrating underutilisation of rehabilitation specifically for children with cancer (50, 51). The reasons for this are multifaceted; however, our patient cohort faced unique challenges that likely worsened the underutilisation of rehabilitation services. Conflict zones may present distinct, complex challenges different from well-established reasons for underutilisation and may, in turn, exacerbate them.

Most of this cohort – over 80%- were evacuated out of Gaza due to their acute need for definitive oncological diagnosis and treatment. Though timeframes vary significantly, some children were evacuated within a few weeks to months of being registered with CNN. With the initial focus understandably being on diagnosis and treatment, and coordinating evacuation, the opportunity for referral and access to rehabilitation may have been significantly limited, and rehabilitation involvement may not have been appropriate at that stage. Due to post-evacuation care being provided by other organisations or organised by the relevant host countries, CNN had limited information on the progress of evacuated children, but it is possible they engaged in rehabilitation intervention abroad.

For the children with cancer in our cohort who remained in Gaza, there were several factors that likely impacted their access to and ability to engage in rehabilitation and physical activity. These include:

#### Loss of health and rehabilitation infrastructure and staff

Enhancing access to rehabilitation services throughout the entire care process is crucial for improving long-term outcomes for children with cancer (8); however, armed conflict harms health systems, including rehabilitation services, and hinders access (52). There are often gaps in ongoing rehabilitation programs and long-term resources that support children with cancer in staying physically active (53); however, the situation in Gaza presented unique challenges, such as the decimation of Gaza’s rehabilitation infrastructure and workforce (16), that likely exacerbated the gaps our patient cohort faced.

The effectiveness of rehabilitation programs for children with cancer depends on a comprehensive and multidisciplinary approach (8, 54); however, in many low- and middle-income countries, including Palestine (55), there is already a shortage of skilled rehabilitation practitioners (49). The conflict-related depletion and displacement of the rehabilitation workforce in Gaza (16) likely exacerbate this existing challenge. Furthermore, workforce limitations make an effective response to rehabilitative needs during conflict and humanitarian crises extremely challenging (52).

#### Unavailability of equipment

Rehabilitation equipment such as crutches, walking aids, wheelchairs, toilet chairs and prosthetics is in very limited supply in Gaza (15, 28) and is among the medical supplies barred by Israel from entering Gaza (56). CNN rehabilitation teams were unable to provide wheelchairs, mobility equipment, orthotics and prosthetics to all but a few children in Gaza during the conflict due to exhausted stocks and huge demand (15, 16). The unavailability of this equipment likely led to reduced independence and greater reliance on caregivers, and undermined the supportive framework children require to participate fully in rehabilitation.

#### Destruction of general infrastructure and displacement

Environmental and economic barriers to accessing rehabilitation may also contribute to underutilisation. Destroyed roads, transport, and other transport infrastructure pose a significant barrier to families’ access to medical and health services (57). Some children with mobility issues had no access to wheelchairs or mobility aids due to a lack of availability (16) and the Israeli blockade of medical equipment (15), creating additional transport challenges.

During the conflict, 90% of Gaza’s population was displaced, many forcibly so, which affected more than one million children (58). Displacement often happened repeatedly, with healthcare professionals at CNN reporting that displacement occurred nine times for some during the conflict, and that some were unable to return home even after the announced ceasefire (personal communication, Children Not Numbers, Gaza Rehabilitation Lead. October, 2025).

#### Malnutrition and lack of analgesic medications

Famine in Gaza, characterised by widespread starvation (59), led to a severe malnutrition crisis (60), which is especially dangerous for children due to their higher nutrient needs for growth and activity (61). Malnutrition during childhood cancer treatment affects clinical outcomes, quality of life, and is linked to reduced physical and social functioning (62). Half of the children with cancer registered with CNN were either malnourished or at risk, which may have negatively impacted their physical function, activity levels (61, 62) and ability to participate in rehabilitation.

Our cohort likely experienced an absence of pain-relief medications (29, 48), which often complement rehabilitation. Together, these factors can be barriers to participation in rehabilitation and physical activity, and consequently, referrals.

#### Referral criteria and awareness of cancer rehabilitation

Though referral criteria for *rehabilitation* based on symptoms and needs exist, the therapeutic use of *physical activity*, regardless of symptoms and needs, was not an explicit criterion. Although physical activity is considered an appropriate intervention in paediatric cancer (7), the lack of a clear referral criterion may have inhibited referrals for this intervention.

Underutilisation of rehabilitation services can be attributed to a lack of awareness or education about cancer rehabilitation among healthcare professionals (63); however, in our cohort, the author team did not perceive this to be a significant issue. Retrospective explanations from MDT clinicians about why these children were not referred for rehabilitation provide some insight into the unique challenges faced by children with cancer in Gaza:

“*Many children [with cancer in Gaza] will not have been referred for rehabilitation due to the severity of their illness and associated medical needs. Some were often too weak or medically unstable because of the lack of adequate treatment and nutrition to participate in or adhere to such interventions.”*

(Triaging Paediatric Doctor, Children Not Numbers.)

“*Rehabilitation is an essential part of the management of childhood cancer care under normal circumstances; however, children and parents faced with a diagnosis of cancer were forced to spend all their time and efforts on simply staying alive and finding a path out of Gaza. Seeking available rehab services was often a journey too dangerous or uncertain to be considered.”*

(Paediatric Oncologist, Children Not Numbers.)

#### Multifaceted consequences of armed conflict

Besides direct challenges to rehabilitation during the conflict, children with cancer in Gaza faced numerous physical, psychological, and social issues, alongside their families’ own precarious and hazardous living conditions.

Armed conflict causes significant suffering for children, and despite legal protections under international humanitarian law, they are often displaced, separated from families, or injured and killed (18). Furthermore, armed conflict impacts heavily on education (18), with over 90% of Gaza’s schools damaged or destroyed (64) and six hundred thousand of Gaza’s 658,000 school-aged children not receiving education for 2 years during the conflict (65).

Over 39,000 children in Gaza have lost one or both of their parents, and of these, 17,000 have lost both their parents (66). It is well-documented that parents play a key role in their child’s cancer and rehabilitation journey (67, 68), so this is likely to have had a detrimental effect on access to care for children with health and rehabilitation needs.

The records we had available did not document the psychological impact of conflict or its effects on education; however, those effects on children in Gaza are well documented. Studies have shown that children with cancer in Gaza and their families have experienced a significant emotional toll from both the illness and the effect of the conflict (26), and we can assume our cohort experienced similar impacts. The conflict in Gaza has significantly affected children’s mental health and psychological well-being (69), which is likely to adversely influence their ability to engage with rehabilitation. Mental health and psychosocial support should be integrated and expanded in parallel with rehabilitation efforts for children in Gaza (45).

### Rehabilitation and physical activity

Many children with cancer in Gaza had a documented rehabilitation-related issue or symptoms such as pain, fatigue, weakness and swallowing difficulties. These children could have benefited from rehabilitation services, including physiotherapy, occupational therapy and speech and language therapy. Based on diagnostic or age groups, other children were also likely to have benefited from rehabilitation-related assessment and intervention. With over 335,000 children under five years in Gaza facing a high risk of severe developmental delays due to the collapse of early childhood services (64), the 33% of children in our cohort in that age bracket might have benefited from developmental screening to identify and address developmental delays or other functional impairments. This is particularly important in conflict zones, where exposure to conflict increases the likelihood of delayed early childhood development, especially with long-term exposure (70).

Although physical activity needs could not be identified from the medical records of this cohort, many children may have benefited from some level of physical activity and exercise intervention to mitigate cancer-related symptoms and reduce the effects of deconditioning (7).

Despite the benefits of physical activity for children (71) and those with cancer (7, 53, 72), over 80% of young people worldwide do not meet recommended activity levels (71), and specific physical activity programs for children with cancer are rare (73). Studies in non-conflict settings show parents of children with cancer are motivated but limited in overcoming barriers to physical activity, highlighting the need for comprehensive support from the treatment organisation (74). In Gaza, care was already fragmented (34), and much of the rehabilitation infrastructure has been damaged or destroyed (16), hindering available professional support.

However, addressing physical inactivity in children with cancer requires not only children and parents but also environmental support to promote activity (74). Gaza’s war conditions pose significant environmental challenges for children, including those with cancer, in maintaining recommended physical activity levels safely. Besides over 80% of Gaza’s buildings and infrastructure being destroyed, four million tons of rubble containing toxic material and chemical waste (75), along with unexploded ordnance (76), pose a serious ongoing threat to children. Most child-friendly spaces in northern Gaza are closed or inaccessible (15), and sewage contamination, air pollution, and vermin and insect infestations (75) pose health risks and hinder children’s outdoor activity and play in Gaza. Lack of physical activity harms not only children’s physical and mental health but also strains health services and society as a whole (71).

## Conclusion

The experience of the CNN rehabilitation team during the current conflict in Gaza has demonstrated an underutilisation of rehabilitation services in children with suspected or confirmed cancer, where cancer-related symptoms, developmental delays, and functional impairments may have been alleviated through physiotherapy, occupational therapy, speech and language therapy, and other rehabilitation disciplines. It is vital to highlight and understand the reasons behind this service gap. We intend to investigate further and identify how this gap can be closed to most effectively support these vulnerable children.

Beyond the context of CNN, other organisations operating in conflict zones should consider referring all children with conditions for which rehabilitation may alleviate debilitating symptoms or improve an impairment in function to appropriate services, regardless of the conflict context or the perceived importance or relative priority of rehabilitation interventions. Clear referral criteria, including specifying referrals for physical activity and exercise interventions for children with cancer, and proactively encouraging referrals from diagnostic or other groups with low referral rates, may facilitate more referrals to rehabilitation. Proactively highlighting and promoting to the wider MDT what rehabilitation interventions can be provided for children with cancer - including equipment and other assistive technology, medications, and multi-disciplinary therapy such as physiotherapy, occupational therapy and speech and language therapy - can assist clinicians in considering which patients to refer to rehabilitation services.

Further discussion and research are needed on whether, how, and what types of non–war–related rehabilitation should be prioritised, and on how it can be delivered in conflict zones.

However, of more immediate priority is ensuring the protection of healthcare facilities and infrastructure and the timely and unimpeded evacuation of children with cancer, in accordance with international humanitarian law (77), so they receive appropriate cancer diagnosis and treatment, as well as holistic care, including rehabilitation. In regions of armed conflict, protection of medical and healthcare workers and infrastructure, as outlined under international humanitarian law (77), is paramount to maintaining healthcare and rehabilitation services for children with cancer who are unable to be evacuated.

Reasons for the lack of referrals for rehabilitation, which consequently led to the absence of rehabilitation-related data, require further understanding, especially to enable appropriate changes to be considered. Studies to explore why clinicians are not referring children in conflict zones, or whether families would potentially engage with intervention, are vitally needed.

Despite the context of conflict, ensuring that holistic needs, including rehabilitation, are identified and comprehensively documented would improve understanding of this cohort’s needs and potentially increase referral rates to appropriate services. Future directions in providing rehabilitation services in conflict zones should consider how this is balanced against other health care and survival needs.

The current situation provides an opportunity to develop a suitable level of specialist and dedicated paediatric oncology rehabilitation and physical medicine services, including education and training, as part of the broader rebuilding and necessary expansion of Gaza’s rehabilitation infrastructure (16) and previously fragmented health services (34).

To our understanding, no studies exist on the rehabilitation and physical activity needs and challenges of children with cancer in conflict settings. Over half a billion children are currently living within a conflict zone, which equates to almost one-fifth of the world’s population of children (78). It is imperative, therefore, that the rehabilitation and physical activity needs of children with cancer in conflict zones, as well as barriers to the provision of services, are better understood so that their holistic care can be optimised.

## Acknowledgment of conceptual or methodological constraints

The primary limitation of this report is the limited availability of data on the rehabilitation and physical activity needs and challenges of children with cancer in conflict settings. Furthermore, the absence of referrals for rehabilitation and the sparse, inconsistent recording of rehabilitation-related information in medical records contributed to the lack of data, restricting the conclusions that could be drawn about the rehabilitation needs of this group of children. Had the CNN rehabilitation team assessed these children, it is likely that more comprehensive and holistic information about their symptoms and needs would have been identified.

These circumstances, however, reflected the limited protocols and procedures available to a newly established NGO and its workforce, as well as the NGO’s evolution amid a complex and evolving contemporaneous conflict.

The paucity of literature on paediatric oncology care, including rehabilitation, in both Gaza and conflict zones more widely, led the authors to seek grey literature, which, while providing a firsthand account of events on the ground, has not been peer-reviewed. Although we conducted a thorough search of the grey literature, some relevant publications may have been omitted. Furthermore, our searches were limited to English-language reports.

Given that this is a single NGO’s experience, findings may not be generalisable to other settings or organisations.

## Data Availability

The data analyzed in this study is subject to the following licenses/restrictions: Patient identifiable, confidential and sensitive data. Requests to access these datasets should be directed to abu.sidhanee@childrennotnumbers.org.

## References

[1] Rossi F Valle M Carlucci G Tofani M Galeoto G Berchialla P. Development of functional abilities assessment in paediatric oncology (FAAP-O) scale for children and adolescents affected by cancer. Children (Basel). (2025) 12(9):1163. 10.3390/children12091163 PMID: 41007028; PMCID: PMC12468092. 41007028 PMC12468092

[2] Grimshaw SL Conyers R van Dalen EC Ness K Verwaaijen EJ. Establishing consensus on defining the physically vulnerable child with cancer: a protocol for an international Delphi approach. BMJ Paediatr Open. (2025) 9(1):e003401. 10.1136/bmjpo-2025-003401 PMID: 40541284 PMCID: PMC12182134. 40541284 PMC12182134

[3] Ospina PA McNeely ML. A scoping review of physical therapy interventions for childhood cancers. Physiother Can. (2019) 71(3):287–96. 10.3138/ptc.2018-13.pp PMID: 31719724 PMCID: PMC6830414. 31719724 PMC6830414

[4] Wright M Gorter M. The “F-words” in pediatric oncology: improving pediatric cancer care through innovative thinking and rehabilitation for optimal quality of life. Rehabil Oncol. (2025) 43(1):2–9. 10.1097/01.REO.0000000000000379

[5] Pruitt DW Haas MT Bolikal PD. Functional impairment in pediatric cancer survivorship. Pediatr Clin North Am. (2023) 70(3):501–15. 10.1016/j.pcl.2023.01.002 PMID: 37121639. 37121639

[6] Mitra R. Principles of rehabilitation medicine. McGraw-Hill Education. (2019). Available online at: https://neurology.mhmedical.com/book.aspx?bookid=2550 (Accessed February 5, 2026).

[7] Braam K van der Torre P Takken T Veening M van Dulmen-den Broeder E Kaspers G. Physical exercise training interventions for children and young adults during and after treatment for childhood cancer. Cochrane Database Syst Rev. (2016) (3):CD008796. 10.1002/14651858.CD008796.pub3 27030386 PMC6464400

[8] L’Hotta AJ Beam IA Thomas KM. Development of a comprehensive pediatric oncology rehabilitation program. Pediatr Blood Cancer. (2020) 67(2):e28083. 10.1002/pbc.28083 31736277

[9] Ospina PA Wiart L Eisenstat DD McNeely ML. Physical rehabilitation practices for children and adolescents with cancer in Canada. Physiotherapy Canada. (2020) 72(2):207–16. 10.3138/ptc-2018-0077 32494104 PMC7238940

[10] Palestinian Central Bureau of Statistics. Palestine: Palestinian Central Bureau of Statistics. (2022). Available online at: https://www.pcbs.gov.ps/portals/_pcbs/PressRelease/Press_En_ChildDayE2022.pdf (Accessed February 17, 2026).

[11] Palestinian Central Bureau of Statistics. Palestinian Central Bureau of Statistics (PCBS) presents the conditions of Palestinian populations on the occasion of the International Population Day, 11/07/2022. Palestinian Central Bureau of Statistics (PCBS). Available online at: https://pcbs.gov.ps/post.aspx?lang=en&ItemID=4279 (Accessed February 14, 2026).

[12] United Nations Human Rights Office of the High Commissioner. Israel has committed genocide in the Gaza Strip, UN Commission finds. Available online at: https://www.ohchr.org/en/press-releases/2025/09/israel-has-committed-genocide-gaza-strip-un-commission-finds (Accessed March 22, 2026).

[13] Amnesty International. Amnesty International concludes Israel is committing genocide against Palestinians in Gaza. (2024). Available online at: https://www.amnesty.org/en/latest/news/2024/12/amnesty-international-concludes-israel-is-committing-genocide-against-palestinians-in-gaza/ (Accessed February 2, 2026).

[14] European Public Health Alliance. Joint Public Health Statement on Gaza. Available online at: https://epha.org/joint-public-health-statement-on-gaza/ (Accessed February 4, 2026).

[15] United Nations Office for the Coordination of Humanitarian Affairs - Occupied Palestinian Territory. Humanitarian situation update #329 | Gaza Strip. (2025). Available online at: https://www.ochaopt.org/content/humanitarian-situation-update-329-gaza-strip (Accessed October 8, 2025)

[16] World Health Organization. Estimating trauma rehabilitation needs in Gaza using injury data from emergency medical teams. Available online at: https://www.who.int/publications/m/item/estimating-trauma-rehabilitation-needs-in-gaza-using-injury-data-from-emergency-medical-teams (Accessed February 9, 2026).

[17] Ali M Rehman IU Lee KS Ming LC. Gaza’s health emergency: impact of armed conflict and its global health repercussions. Global Health. (2025) 21(1):65. 10.1186/s12992-025-01161-0 41194183 PMC12590689

[18] International Committee of the Red Cross. Protected persons: children. (2014). Available online at: https://www.icrc.org/en/law-and-policy/protected-persons-children (Accessed January 27, 2026).

[19] Mohammad L. Children make up nearly half of Gaza’s population. Here’s what it means for the war. NPR. (2023). Available online at: https://www.npr.org/2023/10/19/1206479861/israel-gaza-hamas-children-population-war-palestinians (Accessed October 7, 2024).

[20] Save the Children. Gaza: 20,000 children killed in 23 months of war - more than one child killed every hour. (2025). Available online at: https://www.savethechildren.net/news/gaza-20000-children-killed-23-months-war-more-one-child-killed-every-hour (Accessed January 29, 2026).

[21] Oxfam International. More women and children killed in Gaza by Israeli military than any other recent conflict in a single year. Oxfam International. (2025). Available online at: https://www.oxfam.org/en/press-releases/more-women-and-children-killed-gaza-israeli-military-any-other-recent-conflict (Accessed October 7, 2024).

[22] UNICEF. Children disproportionately wearing the scars of the war in Gaza - Geneva Palais briefing note. (2024). Available online at: https://www.unicef.org/press-releases/children-disproportionately-wearing-scars-war-gaza-geneva-palais-briefing-note (Accessed January 29, 2026).

[23] Khatib R McKee M Yusuf S. Counting the dead in gaza: difficult but essential. Lancet. (2024) 404(10449):237–8. 10.1016/S0140-6736(24)01169-3 38976995

[24] UNICEF. During Gaza’s ceasefire, children keep being killed. (2026). Available online at: https://www.unicef.org/press-releases/during-gazas-ceasefire-children-keep-being-killed (Accessed January 27, 2026)

[25] United Nations Office for the Coordination of Humanitarian Affairs - Occupied Palestinian Territory. Humanitarian situation report, 19 March 2026. (2026). Available online at: https://www.ochaopt.org/content/humanitarian-situation-report-19-march-2026 (Accessed March 26, 2026)

[26] Rihani R Jeha S Salman Z Amer S Qaddoumi I Rodriguez-Galindo C Mansour A. Restoring health and hope to displaced Gaza children with malignant disease at a cancer centre in Jordan. Available online at: https://www.emro.who.int/emhj-volume-31-2025/volume-31-issue-4/restoring-health-and-hope-to-displaced-gaza-children-with-malignant-disease-at-a-cancer-centre-in-jordan.html (Accessed January 29, 2026). 10.26719/2025.31.4.243 40448488

[27] Salman Z Shbair M Zeineddin M Balousha T Qaddoumi I Rodriguez-Galindo C. Cancer care for children in the Gaza Strip. Lancet Oncol. (2021) 22(12):1667–8. 10.1016/S1470-2045(21)00655-0 34856140

[28] Rimawi R Wispelwey B Madani N. Roadblocks to cancer care in the occupied Palestinian Territories. Health Hum Rights. (2024) 26:39–44. PubMed PMID: 39742203 PubMed Central PMCID: PMC11683580. https://pmc.ncbi.nlm.nih.gov/articles/PMC11683580/39742203 PMC11683580

[29] Tondo L Tantesh S Taha S. ‘I wish I had the power to ease his suffering’: Gaza’s cancer patients trapped by war and blockade. The Guardian. (2026). Available online at: https://www.theguardian.com/world/2026/jan/24/i-wish-i-had-the-power-to-ease-his-suffering-gazas-cancer-patients-trapped-by-war-and-blockade (Accessed January 26, 2026)

[30] Heaney C. Right to health barriers to health and attacks on health care in the OPT, 2019 to 2021 - WHO Report. Question of Palestine. Available online at: https://www.un.org/unispal/document/right-to-health-barriers-to-health-and-attacks-on-health-care-in-the-opt-2019-to-2021-who-report/ (Accessed February 8, 2026).

[31] Rimawi R Carpenter K Taleb W Lehmann L Saleh H Madani N. Disparities in pediatric oncology access and outcomes in the occupied Palestinian territories: a retrospective study from Augusta Victoria Hospital. eClinicalMed. (2025) 88. 10.1016/j.eclinm.2025.103463 PMC12572807 41181833

[32] Save the Children. “I can’t run, play, or get treatment” - 16-year blockade leaves two children a day in Gaza unable to access medical treatment - Save the Children Australia. (2023). Available online at: https://www.savethechildren.org.au/media/media-releases/i-cant-run-play-or-get-treatment (Accessed January 8, 2026).

[33] World Health Organization. Delivering childhood cancer care in fragile and conflict settings: best practices from the occupied Palestine territory. Available online at: https://www.who.int/news-room/feature-stories/detail/delivering-childhood-cancer-care-in-fragile-and-conflict-settings-best-practices-from-palestine (Accessed September 16, 2025).

[34] World Health Organization. Health conditions in the occupied Palestinian territory, including east Jerusalem, and in the occupied Syrian Golan. Report by the Director General. Provisional agenda item 20. The 75 World Health Assembly. (2022). Available online at: https://apps.who.int/gb/ebwha/pdf_files/WHA75/A75_26-en.pdf (Accessed September 16, 2025).

[35] Skelton M Al-Mash’hadani AK Abdul-Sater Z Saleem M Alsaad S Kahtan M. War and oncology: cancer care in five Iraqi provinces impacted by the ISIL conflict. Front Oncol. (2023) 13:1151242. 10.3389/fonc.2023.1151242 37213303 PMC10196689

[36] The International Commission for Supporting Palestinian People’s Rights (ICSPR). ICSPR on World Cancer Day… Gaza’s cancer patients face a slow death under bombardment, siege, and the collapse of the health system - ICSPR. (2026). Available online at: https://en.icspr.ps/icspr-on-world-cancer-day-gazas-cancer-patients-face-a-slow-death-under-bombardment-siege-and-the-collapse-of-the-health-system/6828/ (Accessed February 6, 2026).

[37] Statement on the Forced Closure of PCRF’s Pediatric Cancer Department in Gaza. Palestine children’s relief fund. Available online at: https://www.pcrf.net/pcrf-in-the-news/statement-on-cancer-department-in-gaza.html (Accessed January 31, 2026).

[38] United Nations Office for the Coordination of Humanitarian Affairs. (2026). Humanitarian response | Situation Report No. 64. Available online at: https://www.unocha.org/publications/report/occupied-palestinian-territory/gaza-humanitarian-response-situation-report-no-64 (Accessed April 27, 2026)

[39] Mann I Aburass A Leibowitz T Shalev G. Position Paper: Destruction of conditions of life: a health analysis of the Gaza genocide. Physicians for Human Rights Israel. (2025). Available online at: https://www.phr.org.il/wp-content/uploads/2025/07/Genocide-in-Gaza-PHRI-English.pdf (Accessed February 17, 2026)

[40] Amer LA Ruwaida. Out of medicines, care: Gaza’s cancer patients face death amid Israel war. Al Jazeera. Available online at: https://www.aljazeera.com/news/2023/11/14/out-of-medicines-care-gazas-cancer-patients-face-death-amid-israel-war (Accessed February 5, 2026).

[41] Knell Y Evans J. Gaza children dying as they wait for Israel to enable evacuations. BBC. (2025). Available online at: https://www.bbc.com/news/articles/cze61zg7zzpo (Accessed October 26, 2025).

[42] St. Jude Children’s Research Hospital. (2026). St. Jude Global. Taking St. Jude to the world. Available online at: https://www.stjude.org/global.html (Accessed March 26, 2026)

[43] World Health Organization. The occupied Palestinian territory health cluster. Medical evacuation of Gaza patients. (2026). Available online at: https://app.powerbi.com/view?r=eyJrIjoiODAxNTYzMDYtMjQ3YS00OTMzLTkxMWQtOTU1NWEwMzE5NTMwIiwidCI6ImY2MTBjMGI3LWJkMjQtNGIzOS04MTBiLTNkYzI4MGFmYjU5MCIsImMiOjh9 (Accessed April 27, 2026).

[44] World Health Organization. CureAll framework: WHO global initiative for childhood cancer. (2021). Available online at: https://www.who.int/publications/i/item/9789240025271 (Accessed February 4, 2026)

[45] United Nations. Gaza’s many injured will need rehabilitation care and support for years to come, WHO report. (2025). Available online at: https://www.un.org/unispal/document/who-report-02oct25/ (Accessed March 27, 2026)

[46] Children Not Numbers. Who we are. Available online at: https://childrennotnumbers.org/who-we-are/ (Accessed January 31, 2026).

[47] Pleysier S Ickmans K Malfliet A Wauters A van der Werff ten Bossch J Debulpaep S. Exploring pain and body composition in children with cancer compared to healthy controls: a cross-sectional case-control study. Children. (2025) 12(9):1166. 10.3390/children12091166 41007031 PMC12468649

[48] Mansour M Abu Azzoum T. ‘We just sit and cry’: Gaza’s cancer patients die waiting for treatment. Al Jazeera. (2026). Available online at: https://www.aljazeera.com/news/2026/1/9/we-just-sit-and-cry-gazas-cancer-patients-die-waiting-for-treatment (Accessed February 5, 2026)

[49] World Health Organization. Rehabilitation 2030 initiative. (2023). Available online at: https://www.who.int/initiatives/rehabilitation-2030 (Accessed February 17, 2026)

[50] Marchese V Savage L Sun K Situt M York T Reoli R. Physical therapy is an underutilized health resource for children with cancer: a retrospective study identifies facilitators for improvement. Healthcare. (2026) 14(1):20. 10.3390/healthcare14010020 PMC12785601 41516950

[51] Ospina PA Pritchard L Eisenstat DD McNeely ML. Advancing pediatric oncology rehabilitation: survey findings of health Professionals’ perceptions of barriers to care and a framework for action. Cancers (Basel). (2023) 15(3):693. 10.3390/cancers15030693 36765655 PMC9913711

[52] Jain RP Meteke S Gaffey MF Kamali M Munyuzangabo M Als D. Delivering trauma and rehabilitation interventions to women and children in conflict settings: a systematic review. BMJ Glob Health. (2020) 5(Suppl 1):e001980. 10.1136/bmjgh-2019-001980 32399262 PMC7204922

[53] Piscione PJ Bouffet E Mabbott DJ Shams I Kulkarni AV. Physical functioning in pediatric survivors of childhood posterior fossa brain tumors. Neuro-Oncology. (2014) 16(1):147–55. 10.1093/neuonc/not138 24305707 PMC3870837

[54] Tanner LR Sencer S Gossai N Watson D Hooke MC. CREATE childhood cancer rehabilitation program development: increase access through interprofessional collaboration. Pediatr Blood Cancer. (2022) 69(11):e29912. 10.1002/pbc.29912 35986689

[55] Hamadeh N Van Rompaey C Metreau E. World Bank Group country classifications by income level for FY24 (July 1, 2023- June 30, 2024). World Bank Blogs. (2023) Available online at: https://blogs.worldbank.org/en/opendata/new-world-bank-group-country-classifications-income-level-fy24 (Accessed October 7, 2024).

[56] Qiblawi T. CNN. (2024). Anesthetics, crutches, dates: The aid Israel is arbitrarily keeping from Gaza. Available online at: https://www.cnn.com/2024/03/01/middleeast/gaza-aid-israel-restrictions-investigation-intl-cmd (Accessed January 8, 2026)

[57] Doctors Without Borders. How a year of war has devastated Gaza’s civilian infrastructure. (2024). Available online at: https://www.doctorswithoutborders.org/latest/how-year-war-has-devastated-gazas-civilian-infrastructure (Accessed March 22, 2026)

[58] United Nations. World News in Brief: Nearly a million children displaced in Gaza, Guatemala ‘coup’ concerns, ‘enduring menace’ of genocide. (2023). Available online at: https://news.un.org/en/story/2023/12/1144607 (Accessed March 19, 2026)

[59] World Health Organization. Famine confirmed for first time in Gaza. (2025). Available online at: https://www.who.int/news/item/22-08-2025-famine-confirmed-for-first-time-in-gaza (Accessed January 31, 2026)

[60] UNICEF. Two years of hellish war have devastated Gaza’s children. (2025). Available online at: https://www.unicef.org/press-releases/two-years-hellish-war-have-devastated-gazas-children (Accessed October 8, 2025)

[61] Drammeh W Hamid N Jalil R. Determinants of household food insecurity and its association with child malnutrition in Sub-Saharan Africa: a review of the literature. Current Research in Nutrition and Food Science Journal. (2019) 7:610–23. 10.12944/CRNFSJ.7.3.02

[62] Brinksma A Sanderman R Roodbol P Sulkers E Burgerhof J Bont E. Malnutrition is associated with worse health-related quality of life in children with cancer. Support Care Cancer. (2015) 23:3043–52. 10.1007/s00520-015-2674-0 25752883 PMC4552776

[63] Houdeshell MJ Thomas KM King AA L’Hotta AJ. Limitations of current rehabilitation practices in pediatric oncology: implications for improving comprehensive clinical care. Arch Phys Med Rehabil. (2021) 102(12):2353–61. 10.1016/j.apmr.2021.05.021 34339659

[64] UNICEF. Rebuilding hope: UNICEF expands ‘Back to Learning’ for hundreds of thousands of children in Gaza. (2026). Available online at: https://www.unicef.org/press-releases/rebuilding-hope-unicef-expands-back-learning-hundreds-thousands-children-gaza (Accessed March 19, 2026)

[65] Khalil S. Gaza children return to school after years without formal education. BBC. (2026). Available online at: https://www.bbc.com/news/articles/c62vmn30j3yo (Accessed March 22, 2026).

[66] Al Jazeera Staff. Palestinian grandparents care for 36 children orphaned by Israel in Gaza. Al Jazeera. (2025). Available online at: https://www.aljazeera.com/news/2025/10/28/palestinian-grandparents-care-for-36-children-orphaned-by-israel-in-gaza (Accessed February 5, 2026)

[67] Reshetnikov A Gevandova M Prisyazhnaya N Vyatkina N. The role of parents in their child’s cancer diagnosis, treatment, rehabilitation, and socialization. Indian J Pediatr. (2022) 91:30–4. 10.1007/s12098-022-04387-7 36424520

[68] Raber M Swartz MC Santa Maria D O’Connor T Baranowski T Li R. Parental involvement in exercise and diet interventions for childhood cancer survivors: a systematic review. Pediatr Res. (2016) 80(3):338–46. 10.1038/pr.2016.84 27064243

[69] UNICEF. Stories of loss and grief: At least 17,000 children are estimated to be unaccompanied or separated from their parents in the Gaza Strip. (2024). Available online at: https://www.unicef.org/press-releases/stories-loss-and-grief-least-17000-children-are-estimated-be-unaccompanied-or (Accessed January 22, 2026)

[70] Goto R Frodl T Skokauskas N. Armed conflict and early childhood development in 12 low- and middle-income countries. Pediatrics. (2021) 148(3):e2021050332. 10.1542/peds.2021-050332 34404740

[71] World Health Organization. The Global Status Report on physical activity 2022. (2022). Available online at: https://iris.who.int/server/api/core/bitstreams/8804f1b0-dbae-4e58-a251-36fd14dc7e02/content (Accessed February 5, 2026)

[72] The Children & Young People’s Cancer Association. Physical activity and exercise guidelines. (2025). Available online at: https://www.cclg.org.uk/sites/default/files/2026-02/cclg-physical-activity-guideline-sep2025v2.pdf (Accessed March 22, 2026)

[73] Wurz A Daeggelmann J Albinati N Kronlund L Chamorro-Viña C Culos-Reed SN. Physical activity programs for children diagnosed with cancer: an international environmental scan. Support Care Cancer. (2019) 27(4):1153–62. 10.1007/s00520-019-04669-5 30726517

[74] Grimshaw SL Taylor NF Mechinaud F Conyers R Shields N. Physical activity for children undergoing acute cancer treatment: a qualitative study of parental perspectives. Pediatr Blood Cancer. (2020) 67(6):e28264. 10.1002/pbc.28264 32277806

[75] Ben-Shimon D. Gaza faces an environmental catastrophe after war. The Jerusalem Post. (2026). Available online at: https://www.jpost.com/jerusalem-report/article-884620 (Accessed March 22, 2026)

[76] United Nations. Gaza/UNMAS Unexploded Ordnance. (2025). Available online at: https://media.un.org/unifeed/en/asset/d348/d3489754 (Accessed February 6, 2026)

[77] International Committee of the Red Cross. Respecting and Protecting Health Care in Armed Conflicts and in Situations Not Covered by International Humanitarian Law. (2021). Available online at: https://www.icrc.org/sites/default/files/document/file_list/dp_consult_31_hcid_web.pdf (Accessed February 10, 2026).

[78] Save the Children. Stop the War on Children. (2026). Available online at: https://data.stopwaronchildren.org/ (Accessed February 10, 2026)

